# Elucidation of the Flavor Aspects and Flavor-Associated Genomic Regions in Bottle Gourd (*Lagenaria siceraria*) by Metabolomic Analysis and QTL-seq

**DOI:** 10.3390/foods11162450

**Published:** 2022-08-14

**Authors:** Ying Wang, Yanwei Li, Xiaohua Wu, Xinyi Wu, Zishan Feng, Jian Wang, Baogen Wang, Zhongfu Lu, Guojing Li

**Affiliations:** 1 Institute of Vegetables, Zhejiang Academy of Agricultural Sciences, Hangzhou 310021, China; 2 State Key Laboratory for Managing Biotic and Chemical Threats to the Quality and Safety of Agro-Products, Zhejiang Academy of Agricultural Sciences, Hangzhou 310021, China

**Keywords:** amino-acid-related pathway, flavor-associated gene, flavor variation, metabolite profiling

## Abstract

Bottle gourd (*Lagenaria siceraria*) is a commercially important cucurbitaceous vegetable with health-promoting properties whose collections and cultivars differ considerably in their flavor aspects. However, the metabolomic profile related to flavor has not yet been elucidated. In the present study, a comprehensive metabolite analysis revealed the metabolite profile of the strong-flavor collection “J120” and weak-flavor collection “G32”. The major differentially expressed metabolites included carboxylic acids, their derivatives, and organooxygen compounds, which are involved in amino acid biosynthesis and metabolism. QTL-seq was used to identify candidate genomic regions controlling flavor in a MAGIC population comprising 377 elite lines. Three significant genomic regions were identified, and candidate genes likely associated with flavor were screened. Our study provides useful information for understanding the metabolic causes of flavor variation among bottle gourd collections and cultivars. Furthermore, the identified candidate genomic regions may facilitate rational breeding programs to improve bottle gourd quality.

## 1. Introduction

With domestication records dating back 11,000 years, bottle gourd (*Lagenaria siceraria* (Mol.) Standl.) (2n = 2x = 22) is among the oldest plant species of the Cucurbitaceae family used by humans [1,2,3]. Presumably native to Africa, bottle gourd was probably independently domesticated in Africa and Asia [1,4,5]. Bottle gourd is commonly used in traditional medicine owing to its numerous health-related benefits, including diuretic and anticancer effects [6]. Furthermore, bottle gourd seeds reportedly accumulate essential amino acids and oils in some cultivation regions [7]. Generally, a young, tender bottle gourd is a common vegetable with high market value [8,9].

Bottle gourd fruits have a unique flavor when cooked; furthermore, bottle gourd collections and cultivars can significantly differ in flavor aspects. Specifically, free glutamate content was reported to be a key factor conferring umami taste on bottle gourd [9]. However, flavor is a complex trait, and previous studies have only explained the peculiar flavor from one perspective. Thus, to date, no other chemical ingredients or metabolomic profiles have been identified in bottle gourd despite the widespread use of liquid chromatography–tandem mass spectrometry (LC–MS/MS), which has played a crucial role in detecting metabolites in various fruits and vegetables [10,11,12,13,14].

In a previous study, 17 single-nucleotide polymorphisms (SNPs) were identified as being associated with free glutamate, which, as mentioned earlier, is a key factor conferring umami taste on bottle gourd [9]. However, no other information regarding the peculiar flavor of bottle gourd has been reported, and the relevant genetic determinants remain unexplored.

QTL-seq technology, based on the combination of bulked segregant analysis and whole-genome sequencing, provides a facile method for locating QTLs, an approach that has been applied to the study of agronomic traits in rice, cucumber, and tomato [15,16,17]. Therefore, to better understand the unique flavor of bottle gourd, we performed LC–MS/MS metabolite analysis in two materials significantly differing in flavor profiles. These materials were the two parents of a previously constructed multiparent advanced generation inter-cross (MAGIC) population [18]. Additionally, SNPs/QTL responsible for the peculiar flavor of bottle gourd were identified using QTL-seq analysis of strong- and weak-flavor pools selected from the original MAGIC population. Our results explain the reasons and elucidate the molecular mechanism underlying the peculiar flavor of bottle gourd at the metabolomic level, and our study may facilitate the molecular breeding of cultivars with improved organoleptic properties.

## 2. Materials and Methods

### 2.1. Plant Materials

**Figure 1:**
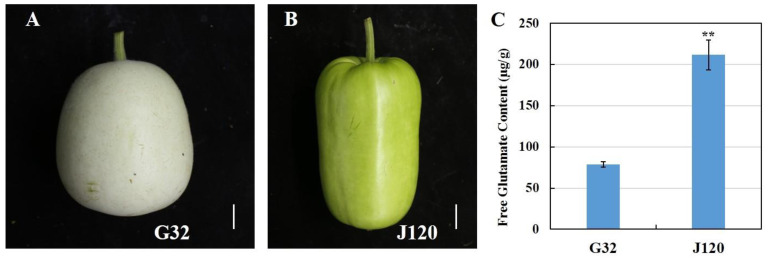
Marketable fruit from “G32” (weak flavor) and “J120” (strong flavor) collections and free glutamate contents. (**A**) Marketable fruit of “G32”, scale bar = 2.1 cm; (**B**) marketable fruit of “J120”, scale bar = 2.5 cm; (**C**) free glutamate content of “G32” and “J120” (** *p* < 0.01).

In this study, we used a previously constructed MAGIC population with a total of 377 MAGIC population elite lines [18] and two parents (“G32” and “J120”, Figure 1) that differed significantly in flavor. These two parents were used for metabolite identification and quantification by LC–MS/MS analysis. The 377 MAGIC population elite lines were used to build strong- and weak-flavor pools for QTL-seq analysis. All accessions were grown in a greenhouse at the Yangdu Scientific Research and Innovation Base of the Zhejiang Academy of Agricultural Sciences (30° N, 120° E). All accessions were cultivated with a distance of 80 cm between the rows and 50 cm between the plants.

### 2.2. Metabolite Extraction

At least six marketable fruits were pooled to create a single biological sample after peeling. Three biological replicates were used for each accession. Samples were freeze-dried using a rotary vane vacuum pump (2XZ-4, Shanghai, China) and then ground to powder for 2 min at 30 Hz in a grinder (MM400, Retsch, Germany) that uses zirconia beads. Subsequently, 100 mg of each sample powder was immersed in 1.2 mL of 70% aqueous methanol overnight at a constant temperature of 4 °C. The sample solutions were centrifuged at 11,000× *g* for 12 min at 4 °C, filtered through a 0.22 μM microporous membrane (SCAA-104, ANPEL, Shanghai, China), and transferred to an LC–MS/MS injection bottle for analysis.

### 2.3. Targeted Metabolomic Analysis

Bottle gourd samples were analyzed using an LC-ESI-MS/MS system (HPLC, Shim-pack UFLC SHIMADZU CBM30A system, MS, Applied Biosystems 4500 Q TRAP, Boston, MA, USA). The chromatographic columns were Waters ACQUITY UPLC HSS T3 C18 columns (1.8 μm, 100 mm × 2.1 mm, Shanghai, China). A 0.04% acetic acid aqueous solution was used as mobile phase A and 0.04% acetic acid acetonitrile as mobile phase B. The gradient was 0 min, 5% B; 5% to 95% B from 0 to 10 min, and maintained at 95% for 1 min; 95% to 5% B from 11 min to 11.1 min, and maintained at 5% to 14 min. The flow rate was 0.35 mL/min. The temperature of the column was 40 °C, and the injection volume was 2 μL. All the reagents were purchased from Merck (Darmstadt, Germany).

The eluent was connected to an ESI triple-quadrupole–linear ion trap (Q TRAP) mass spectrometer (MS) (Q TRAP, Boston, MA, USA) API 4500 Q TRAP LC/MS/MS System equipped with an ESI Turbo Ion-Spray interface, which was operated in positive-ion mode and controlled by Analyst 1.6.3 software (AB Sciex, Framingham, MA, USA). This system was used to acquire linear ion trap (LIT) and triple-quadrupole (QQQ) scans. The ESI source operation parameters were as follows: the ion source was a turbo spray with a 550 °C source temperature; ion-spray voltage was 5500 V; ion source gas I, gas II, and curtain gas were all supplied at 0.2 MPa; and collision-activated dissociation was set at “high”. Polypropylene glycol solutions of 10 and 100 μmol/L were used for instrument tuning and mass calibration in the QQQ and LIT modes, respectively. QQQ scans were acquired in multiple reaction monitoring (MRM) mode, and the collision N_2_ gas was set to 0.35 MPa. The dispersion potential and collision energy were optimized for individual MRM transitions. A specific set of MRM transitions in each period was monitored based on metabolite elution.

### 2.4. Metabolite Identification, Quantification, and Data Analysis

Metabolite identification and quantification were performed as previously described [19]. Metabolites were annotated using a public database of metabolite information. Principal component analysis (PCA)-supervised multiple regression orthogonal partial least-squares discriminant analysis (OPLS-DA), hierarchical cluster analysis, and log2_FC (case_mean/control_mean) were performed using R 4.0.3 [20]. A triple-quadrupole rod was used to detect the characteristic ion of each metabolite. The chromatographic peak of each metabolite was integrated and calibrated with MultiQuant software (SCIEX) and used to estimate the relative content of the corresponding metabolite. To identify the metabolites that accumulated to significantly different extents, metabolite profiles of the strong-flavor collection, “J120” were compared with those of the weak-flavor collection, “G32” Metabolites with a log2_FC > 0 (upregulated) or < 0 (downregulated) in “J120” compared to “G32” were selected and then screened by variable importance in projection (VIP) score ≥ 1.0, and Student’s *t*-test (two-tailed) with a *p* value < 0.05 was used to assess significant differences for metabolite abundance between the two collections. The Kyoto Encyclopedia of Genes and Genomes (KEGG) pathway database was used to determine pathway associations. Pathway enrichment analysis was performed using the web-based server Metabolite Set Enrichment Analysis. 

### 2.5. Strong- and Weak-Flavor Pool Construction

Marketable fruit (8–10 days after pollination) from 377 MAGIC population elite lines were harvested and compared for flavor using a previously published, sensory evaluation method [9]. Briefly, each cooked fruit was evaluated by at least 10 taste panelists. The taste panel consisted of experienced panelists with normal senses of taste and smell. Each panelist independently scored the flavor of each material and recorded the average. The 377 MAGIC population elite lines were divided into different categories according to the intensity of flavor that characterized each line. Finally, strong-flavor elite lines were mixed into a strong-flavor pool, and weak-flavor elite lines were mixed into a weak-flavor pool.

### 2.6. Sample Pool Sequencing and Analysis

A total of four Illumina libraries (the strong- and weak-flavor extreme bulks mentioned above and two parents with significantly contrasting flavors, “G32” and “J120”) were prepared using the Plant Genomic DNA Kit (Qiagen 28304, Beijing, China). Total DNA was quantified using a Qubit 3.0 Fluorometer (Life Technologies, Carlsbad, CA, USA). One microgram of DNA from each sample was used to construct paired-end DNA sequencing libraries using the Illumina TruSeq Nano DNA Sample Prep Kit (Illumina, CA, USA), and the Illumina NovaSeq 6000 platform was used for sequencing.

After trimming and filtering, the short reads obtained from the strong- and weak-flavor pools were mapped against the bottle gourd reference genome [21] using BWA-MEM (bwa mem-k 32) [22]. SNP calling was performed using the Sequence Alignment Map tool [23]. Both the SNP index and the Δ (SNP-index) value [24] for each SNP position were calculated to identify the candidate regions for flavor QTLs. The SNP index is the proportion of reads containing SNPs that are different from the reference sequence. In turn, the Δ (SNP-index) value was calculated by subtracting the SNP index of the strong-flavor from that of the weak-flavor pool. Sliding window analysis, with a 1 Mb window size and 10 kb step increment, was applied to calculate the average of the SNP index. The average SNP-index graphs for the strong- and weak-flavor pools were plotted, as well as the corresponding Δ (SNP-index) graphs. The Δ (SNP-index) value of most genomic regions should be approximately 0, in which genomic regions have no major QTL for the target gene [15].

### 2.7. Statistical Analysis

The data were statistically analyzed using Microsoft Office Excel 2010 (Microsoft, Redmond, WA, USA) and SPSS software (SPSS Inc., Chicago, IL, USA). Data are presented as the mean ± standard error (SE). Student’s *t*-test was used for statistical analyses (* 0.01 ≤ *p* < 0.05,** *p* < 0.01).

## 3. Results and Discussion

### 3.1. Phenotypic Characteristics of “G32” and “J120”

Although planted simultaneously under the same conditions, the two parent lines (“G32” and “J120”) of the previously constructed MAGIC population [18] showed different phenotypic characteristics, particularly in terms of flavor. A previous report showed that free glutamate content is a key factor conferring an umami taste on bottle gourd [9]. Therefore, the free glutamate contents of “G32” and “J120” were assayed before metabolomic analysis. As shown in Figure 1, the free glutamate content differed significantly between the two parent lines. The average free glutamate content of the strong-flavor parent “J120” was 211.8 μg/g, whereas that of its weak-flavor counterpart “G32” was only 78.5 μg/g. Other phenotypic characteristics of the two parents are listed in Table S1. 

Flavor is a complex trait, and free glutamate content is one of the biochemical determinants of flavor. Therefore, the identification and evaluation of bottle gourd flavor at a broader metabolomic level and further identification of the relationship between identified metabolites other than free glutamate are of great significance for the analysis of the main components of gourd fruit flavor and will certainly contribute to breeding efforts aimed at improving fruit quality. Moreover, metabolomics has been previously used to reveal the nutritional aspects of a wide range of food and vegetables [25,26,27].

### 3.2. Metabolite Identification in “G32” and “J120”

**Figure 2:**
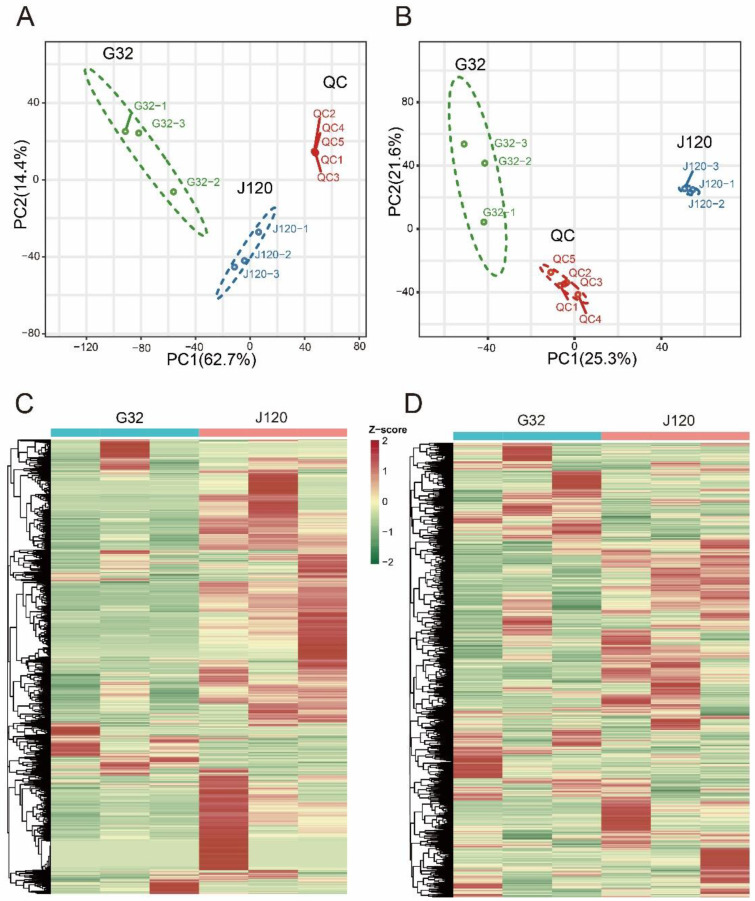
Differential fruit chemotype between “G32” and “J120”. (**A**,**B**): principal components analysis of identified metabolites from “G32” and “J120” in negative ionization (NEG) and positive ionization (POS) modes, respectively. A mixture of equal volumes of “G32” and “J120” samples was used for quality control (QC); (**C**,**D**): cluster analysis of metabolites from “G32” and “J120” fruit in NEG and POS modes, respectively. Colors indicate the level of accumulation of metabolites from low (green) to high (red).

To better understand the flavor differences between “G32” and “J120”, metabolomic analysis was performed to identify the metabolite profiles of these parents that differed significantly in flavor. A total of 1920 metabolites were identified, including 875 negative ionization (NEG) metabolites and 1045 positive ionization (POS) metabolites. PCA was conducted separately under the NEG and POS modes. PCA clearly separated the two materials and quality control samples under both NEG and POS modes (Figure 2A,B). Thus, in the NEG mode, the two principal components, PC1 and PC2, accounted for 62.7% and 14.4% of the total variance, respectively (Figure 2A), whereas in the POS mode, they accounted for 25.3% and 21.6% of the total variance, respectively (Figure 2B). This finding indicates a distinct difference in metabolite composition between “G32” and “J120”.

Subsequently, a log2_FC (case_mean/control_mean) transformation of each metabolite peak area was applied, and hierarchical cluster analysis was subsequently performed in both NEG and POS modes. Two distinct groups were found to be associated with “G32” and “J120” in both NEG and POS modes (Figure 2C,D). Consequently, PCA and hierarchical cluster analysis together suggested that, in addition to their strikingly different flavors, “G32” and “J120” differed significantly in their metabolite profiles. 

**Figure 3:**
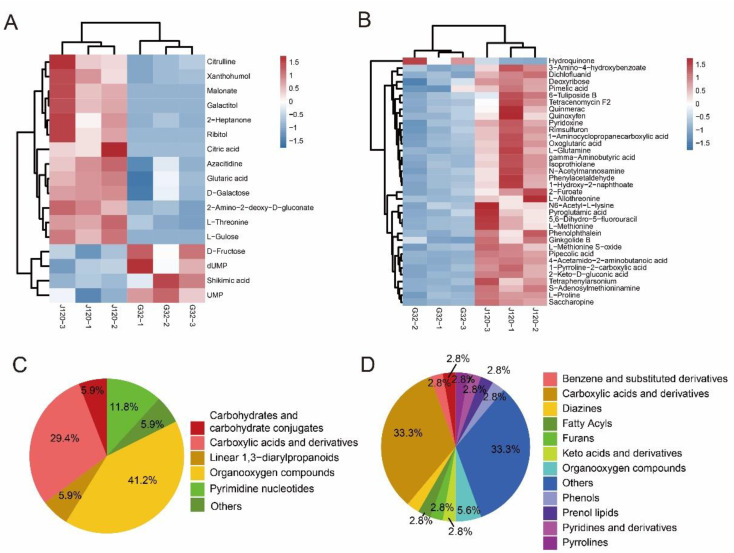
Differentially accumulating metabolites between “G32” and “J120”. (**A**,**B**): identified differential metabolites in “J120” compared to “G32” in negative ionization (NEG) and positive ionization (POS) modes, respectively. (**C**,**D**): pie chart of the differential metabolites identified between “G32” and “J120”.

To identify the differential metabolites between “G32” and “J120”, metabolites with log2_FC > 0 (upregulated) or <0 (downregulated) were selected for comparison between the two collections. A VIP value from the OPLS-DA model was used to screen metabolites with a VIP value ≥ 1.0, followed by a *t*-test at a *p* value < 0.05. Only 17 and 36 differential metabolites were identified between “G32” and “J120” in the NEG and POS modes, respectively (Figure 3B; Tables S2 and S3). Among these, 13 metabolites were upregulated and 4 were downregulated in the NEG mode in “J120” compared to “G32” (Figure 3A). In contrast, in the POS mode, 35 metabolites were upregulated, while only 1 metabolite was downregulated (Figure 3B). The differential metabolites were categorized into different classes under both NEG and POS modes (Figure 3C,D), but the major classes were consistently carboxylic acids, derivatives thereof, and organooxygen compounds (Figure 3C,D; Tables S2 and S3).

### 3.3. KEGG Classification and Enrichment Analysis of Differential Metabolites

**Figure 4:**
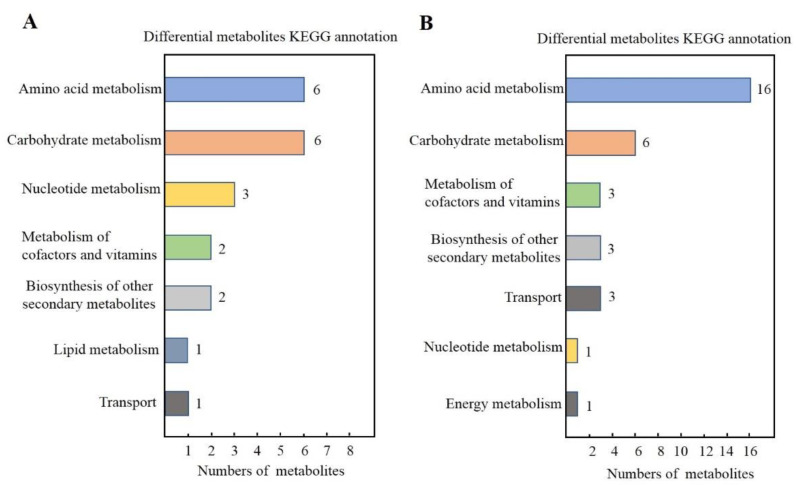
KEGG classification of differential metabolites between “G32” and “J120”. (**A**,**B**): KEGG classification of metabolites differentially accumulated in “J120” and “G32” in NEG and POS modes, respectively.

**Figure 5:**
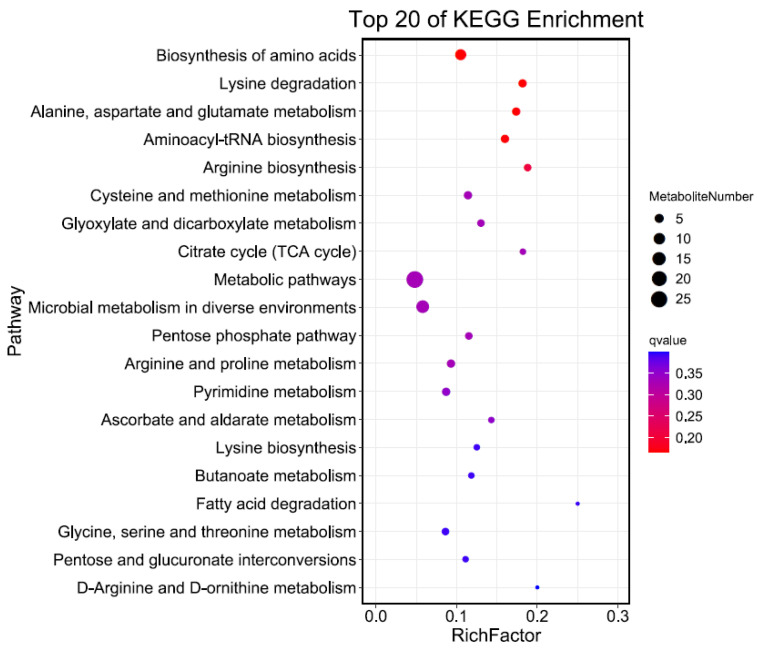
Kyoto Encyclopedia of Genes and Genomes pathway enrichment analysis of differentially accumulated metabolites in “G32” and “J120”.

Seventeen and thirty-six differential metabolites from the “J120” strong- and “G32” weak-flavor parents identified in the NEG and POS modes, respectively, were mapped to the KEGG database. The results show that most of these metabolites were mapped to “Amino acid metabolism”, “Carbohydrate metabolism”, “Nucleotide metabolism”, and “Metabolism of cofactors and vitamins”, and a few of them belonged to other system information categories, such as “Biosynthesis of other secondary metabolites”, “Transport”, and “Energy metabolism” (Figure 4A,B). Subsequently, KEGG pathway enrichment analysis was conducted to identify differences in metabolic pathways between “G32” and “J120”. The enrichment analysis identified metabolites of the amino acid biosynthesis and metabolism pathways (e.g., “Biosynthesis of amino acids”, “Lysine degradation”, and “Alanine, aspartate, and glutamate metabolism”), as different between “G32” and “J120” (Figure 5). Amino acid biosynthesis and metabolism pathways have been previously reported to be major contributors to the flavor of jujube and loquat [28,29]. The significant difference for metabolites involved for amino acid biosynthesis- and metabolism-related pathways between “G32” and “J120” may explain why “J120” tastes better than “G32”. Taste is the result of the synthesis and metabolism of a range of amino acids, not just one.

### 3.4. Extreme Bulks for Flavor

**Figure 6:**
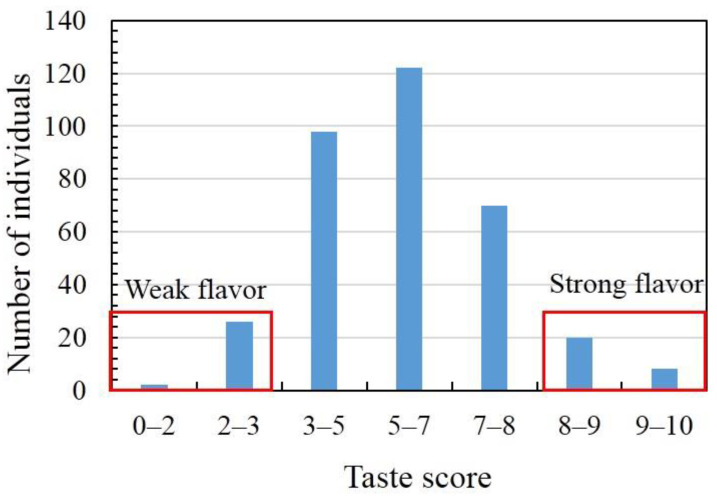
Frequency distribution of taste scores for 346 MAGIC population elite lines of marketable bottle gourd fruit. The DNA of 28 elite lines with extreme phenotypes (high and low taste scores) was selected to prepare strong- and weak-flavor bulks.

A total of 346 MAGIC population elite lines of marketable fruits were harvested and compared for flavor using a previously published sensory evaluation method [9]. After the sensory evaluation of taste, the 346 MAGIC population elite lines were divided into seven categories according to the taste score. As a phenotypic trait, flavor scores recorded by taste panelists showed a nearly normal distribution, with values ranging from 1.80 to 9.43 (Figure 6; Table S4). Consequently, 28 strong-flavor elite lines (taste scores from 8.0 to 9.43) were selected for the strong-flavor pool, and 28 elite lines with a weak flavor (taste scores from 1.80 to 2.97) were selected for the weak-flavor pool (Figure 6). Genotyping only mixed DNA samples from two extreme bulks of individuals with different or opposite extreme phenotypes makes the next-generation-sequencing-based QTL-seq approach a cost-effective, simple, and rapid method for target trait mapping that conveniently avoids tedious genotyping of a large number of populations. Classical QTL mapping is a time-consuming task that includes genotyping the isolated mapping population using polymorphic markers identified between parents and defining important genomic regions of target traits using genotypic and phenotypic data sets [30]. QTL-seq is a successful approach frequently used for identifying target trait genomic regions in cucumber, tomato, and chickpea, among others [16,17,31].

### 3.5. QTL-Seq Allowed the Identification of Three Major QTLs Controlling Flavor

Four Illumina libraries (two for flavor bulks and two for parents) were constructed and subjected to Illumina high-throughput resequencing. The results show 99,923,628, 100,072,612, 107,140,402, and 83,218,116 high-quality clean reads (150 + 150 bp in length) obtained from “G32” (50.3× depth coverage and 93.83% coverage), “J120” (50.5× depth coverage and 93.88% coverage), the strong-flavor line pool (41.9× depth coverage and 98.74% coverage), and the weak-flavor line pool (54.0× depth coverage or 99.03% coverage), respectively (Table 1). A total of 103,275 filtered SNPs were identified between bulks by alignment with the bottle gourd reference genome [21].

**Figure 7:**
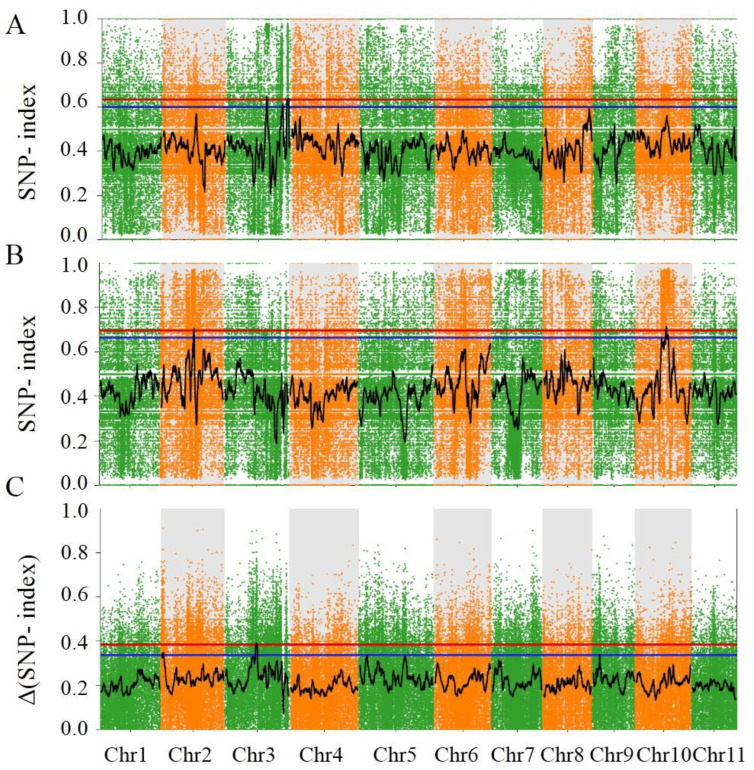
Identification of fruit-flavor-related QTLs in strong- and weak-flavor bulks (**A**–**C**). Single-nucleotide polymorphism (SNP) index plot of strong-flavor bulk, weak-flavor bulk, and ΔSNP-index plot from QTL-seq analysis, respectively. The X-axis represents the position of eleven chromosomes and the Y-axis represents SNP-index values calculated based on 1-Mb intervals with a 10-kb sliding window.

To identify candidate genomic regions presumably responsible for the differences between the strong- and weak-flavor bulks, the SNP index of each identified SNP was calculated and compared. Sliding window analysis with a 1 Mb window size and a 10 kb step increment was applied to calculate the average of the SNP index. In turn, average SNP-index graphs for the strong- and weak-flavor pools were generated (Figure 7A,B) by plotting the SNP index against each sliding window position in the bottle gourd genome. The Δ (SNP index) values were calculated and plotted by subtracting the SNP-index values of the strong-flavor pool from those of the weak-flavor pool (Figure 7C). The SNP-index graphs of the two bulks should be identical, and the genomic regions that are irrelevant to flavor differences and the SNP index of these regions should appear as mirror images to the line of the SNP index = 0.5 [15]. Conversely, a Δ (SNP-index) value different from 0 would indicate that the genomic region harbors a major flavor-related QTL. Our results show that three genomic regions were characterized by a Δ (SNP-index) value significantly different from 0 at a 99.5% level of significance (Figure 7). These regions included chromosome 2 from 90,001 to 1,710,000; chromosome 3 from 13,830,001 to 15,850,000; and chromosome 9 from 2,930,001 to 3,930,000.

In these three genomic regions, 408 putative protein-coding genes were predicted, according to genome annotation (Table S5). Among them, five genes with predicted functions related to amino acids were considered, i.e., interesting candidate genes that might be associated with flavor differences between the two extreme bulks. These included two amino acid permease-coding genes, namely, *HG_GLEAN_10013305* and *HG_GLEAN_10013403*; an amino acid transporter ANT1-encoding gene, *HG_GLEAN_10017438*; and two cationic amino acid transporter-coding genes, i.e., *HG_GLEAN_10017483* and *HG_GLEAN_10017484*. Amino acid permeases are important transmembrane proteins involved in amino acid uptake and transport in plants [32,33]. Amino acid transporters are proteins responsible for amino acid transport and play an important role in plant growth and development [34]. Thus, for example, the expression level of tomato cationic amino acid transporter gene 2 (*SlCAT2*) increased moderately during early fruit development [35]. In addition, amino acid transporter family members may affect grain quality under abiotic stress by regulating amino acid transport and distribution in wheat [36]. Further research is required to elucidate the specific gene functions and relationships with bottle gourd flavor.

## 4. Conclusions

The comprehensive metabolite profile of strong-flavor collection “J120” and weak-flavor collection “G32” has been elucidated. The major differential metabolites were carboxylic acids, their derivatives, and organooxygen compounds involved in amino acid biosynthesis- and metabolism-related pathways. Three flavor-associated genomic regions were identified, and five interesting candidate genes likely associated with bottle gourd flavor were predicted by genome-wide SNP profiling of the extreme flavor bulks from a MAGIC population including 377 bottle gourd elite lines. The results may facilitate rational breeding programs to improve bottle gourd quality.

## Figures and Tables

**Table 1:** Sequencing of parental lines and extreme bulks.

Genotypes	HQ Clean Reads (%)	HQ Clean Bases (bp)	HQ_Q20 (%)	Unmapped Reads (%)	Genome Coverage (%)	Average Depth (X)
G32	99,923,628(99.53%)	14,950,923,292	97.30%	1.82%	93.83%	50.3
J120	100,072,612(99.53%)	14,986,550,841	97.13%	2.28%	93.88%	50.5
Strong-flavor pool	107,140,402(99.48%)	12,448,588,625	96.95%	0.52%	98.74%	41.9
Weak-flavor pool	83,218,116(99.42%)	16,025,817,799	97.16%	0.38%	99.03%	54.0

## Data Availability

The data presented in this study are available on request from the corresponding author.

## Supplementary Materials

The following supporting information can be downloaded at: https://www.mdpi.com/article/10.3390/foods11162450/s1. Table S1: Phenotypic characteristics of “G32” and “J120”; Table S2: Differential metabolites in “J120” compared to “G32”, in the NEG mode; Table S3: Differential metabolites in “J120” compared to “G32”, in the POS mode; Table S4: Taste score of the experimental 346 MAGIC population elite lines; Table S5: Genes in the three selected genomic regions.
